# Salt-Free Diet in the Treatment of Hyperchlorhydria

**Published:** 1913-01

**Authors:** 


Salt-Free Diet in the Treatment of Hyperchlorhydria. M. Leo, (*La Semaine Medicale,* June 12, 1912) noted that the amount of chlorides in the feces of hyperchlorhydrics was below normal, and concluded that the chloride content of the tissues must be above normal. The correctness of this assumption was proved by the fact that a salt-free diet was only followed by a reduction of the free hydrochloric acid in the gastric juice after about two weeks, while in the normal subject the acid diminished almost immediately.

Following the publication of Leo's article, Richartz, of Hamburg, resorted to the salt-free diet in an aggravated case of hyperchlorhydria, in a man sixty years of age, which had proved rebellious to all forms of treatment. The pain was practically constant and enormous doses of alkalies afforded only transitory relief.

In addition to the salt-free diet, which included abstinence from all mineral waters, daily lavage was practiced two hours and a half after the principal meal.

Richartz has since applied this treatment in eight other cases, with similar results in six. He thinks, however, that the failure in the two cases was due to the fact that the patients did not adhere faithfully to the regime. In fact, except with intelligent patients, it is only when the pain is intolerable, that they can keep up the diet for a sufficiently long period. He calls attention to sodium bromide, which is an excellent substitute for salt in rendering the food palatable and, in addition, exercises a sedative action, which is very desirable.

He is of the opinion that the salt-free diet alone cannot bring about the desired result in severe cases, but that the mechanic removal of the excessive acid by lavage is a necessary part of the treatment.

